# LncRNA FOXD1-AS1 regulates pancreatic cancer stem cell properties and 5-FU resistance by regulating the miR-570-3p/SPP1 axis as a ceRNA

**DOI:** 10.1186/s12935-023-03181-5

**Published:** 2024-01-02

**Authors:** Liu Ouyang, Min-min Sun, Ping-sheng Zhou, Yi-wei Ren, Xin-yu Liu, Wan-ying Wei, Zhen-shun Song, Kai Lu, Li-xue Yang

**Affiliations:** 1 Department of Hepatobiliary and Pancreatic (HBP) Surgery, Shanghai Fourth People’s Hospital, School of Medicine, Tongji University, Shanghai, 200434 China; 2 Department of Biliary Tract Surgery II, The Third Affiliated Hospital of Naval Medical University, Shanghai, 200438 China; 3 Department of Hepatic Surgery I, The Third Affiliated Hospital of Naval Medical University, Shanghai, 200438 China; 4 Department of Ultrasonic Intervention, The Third Affiliated Hospital of Naval Medical University, Shanghai, 200438 China; 5 Department of Hepatobiliary and Pancreatic (HBP) Surgery, Changhai Hospital, Naval Medical University, Shanghai, 200433 China

**Keywords:** Pancreatic cancer, Cancer stem cell, lncRNA FOXD1-AS1, N6-methyladenosine, 5-FU

## Abstract

Cancer stem cells (CSCs) play a pivotal role in the pathogenesis of human cancers. Previous studies have highlighted the role of long non-coding RNA (lncRNA) in modulating the stemness of CSCs. In our investigation, we identified an upregulation of lncRNA FOXD1-AS1 in CSCs. The enforced expression of lncRNA FOXD1-AS1 promotes tumorigenesis and self-renewal in pancreatic cancer CSCs. Conversely, the knockdown of lncRNA FOXD1-AS1 inhibits tumorigenesis and self-renewal in pancreatic cancer CSCs. Furthermore, our findings reveal that lncRNA FOXD1-AS1 enhances self-renewal and tumorigenesis in pancreatic cancer CSCs by up-regulating osteopontin/secreted phosphoprotein 1(SPP1) and acting as a ceRNA to sponge miR-570-3p in pancreatic cancer (PC) CSCs. Additionally, lncRNA FOXD1-AS1 depleted pancreatic cancer cells exhibit heightened sensitivity to 5-FU-indued cell growth inhibition and apoptosis. Analysis of patient-derived xenografts (PDX) indicates that a low level of lncRNA FOXD1-AS1 may serve as a predictor of 5-FU benefits in PC patients. Moreover, the introduction of SPP1 can reverse the sensitivity of lncRNA FOXD1-AS1-knockdown PC cells to 5-FU-induced cell apoptosis. Importantly, molecular studies have indicated that the elevated levels of lncRNAFOXD1-AS1 in PC are facilitated through METTL3 and YTHDF1-dependent m^6^A methylation. In summary, our results underscore the critical functions of lncRNA FOXD1-AS1 in the self-renewal and tumorigenesis of pancreatic cancer CSCs, positioning lncRNA FOXD1-AS1 as a promising therapeutic target for PC.

**Supplementary Information:**

The online version contains supplementary material available at 10.1186/s12935-023-03181-5.

## Introduction

Pancreatic cancer (PC) is a major contributor to global-cancer mortality [1]. Surgical excision stands out as the most effective method for early PC diagnosis [2]. Unfortunately, a substantial number of individuals receive a diagnosis at the intermediate or advanced stages of the disease [3]. 5-Among the initial chemotherapy agents for PC treatment, 5-Fluorouracil (5-FU) holds a prominent position. However, the emergence of drug resistance to 5-FU has become a prevalent clinical challenge, significantly compromising the efficacy of chemotherapy. This resistance is a critical determinant affecting therapeutic outcomes in this context [4]. Hence, it is critical to comprehend the molecular mechanisms that drive the aggressive nature and high metastatic potential of 5-FU resistance in pancreatic cancer and to identify and develop innovative treatment targets [5, 6].

Tumor-initiating cells (T-ICs) or Cancer stem cells (CSCs) constitute a distinct subset of cancer cells endowed with the unique capacity for self-renewal and unlimited proliferation [7, 8]. Compelling evidence underscores the pivotal role of CSCs in orchestrating various critical processes, including tumor initiation, proliferation, metastasis, recurrence, and chemoresistance [9–11]. CSCs emerge as decisive factors in tumor metastasis and replication [12, 13]. Previous research has corroborated the existence of pancreatic cancer CSCs [14]. However, the precise mechanism governing the dissemination of pancreatic cancer CSCs remains largely unknown.

Long non-coding RNAs (lncRNAs) are RNA molecules exceeding 200 nucleotides in length that do not encode proteins [15]. Several investigations have provided compelling evidence of the involvement of lncRNAs in regulating diverse biological functions [16]. These molecules can act as either oncogenes or tumor suppressors in various cancers [17–19]. Notably, reports suggest that lncRNA FOXD1-AS1 plays a contributory role in the development of certain solid tumors [20, 21]. However, the potential role of lncRNA FOXD1-AS1 in PC and CSCs remains unknown. In this study, we uncovered elevated levels of lncRNA FOXD1-AS1 in pancreatic cancer CSCs. Moreover, we demonstrated that lncRNA FOXD1-AS1 enhances the self-renewal and tumorigenesis of PC CSCs by regulating the miR-570-3p/SPP1 axis as a ceRNA both in vitro and in vivo. Importantly, lncRNA FOXD1-AS1 emerged as a determinant of the response to 5-FU in PC. Consequently, these findings suggest that lncRNA FOXD1-AS1 may serve as a potential biomarker for the effectiveness of 5-FU therapy and present an exciting avenue for therapeutic interventions in the context of PC.

## Materials and methods

### PC patient tissue

A cohort comprising 30 PC tissues was collected from individuals who underwent surgical procedures at Changhai Hospital between 2008 and 2021 (Additional file 1: Table S1). Patient informed consent was obtained, and the study protocols received approval from the Ethics Committee of The Third Affiliated Hospital of Naval Medical University China.

### Cell culture

The PC cell lines AsPC-1 and MIA Paca-2 were cultured in Dulbecco's modified Eagle's medium (DMEM) supplemented with 25 µg/ml gentamicin, 2 mM L-glutamine, and 10% fetal bovine serum (FBS). These cells were maintained at 37 °C in a 5% CO_2_ incubator. Lentiviruses carrying either lncRNA FOXD1-AS1 knockdown or lncRNA FOXD1-AS1 overexpression constructs, along with control lentiviruses obtained from Ribobio (Shanghai, China), were used to infect MIA Paca-2 and AsPC-1 cell lines. The final transfected cells were selected using puromycin. To establish cell lines resistant to 5-fluorouracil, MIA Paca-2, and AsPC-1 cells underwent incubation with 5-FU at a concentration slightly lower than their half-maximal inhibitory concentration (IC50). The concentration of 5-FU was incrementally increased by 0.2 μM/L each week over a period of 6 to 7 months. Three cell lines demonstrating resistance to 5-FU were obtained and then cultured continuously under exposure to 5-FU to maintain their resistance.

### RNA interference

Ribobio developed siRNAs targeting NC (negative control) and SPP1. The siRNAs, at a concentration of 200 nM, were introduced into PC cells using a siRNA transfection reagent following the manufacturer’s instructions (Polyplus, Illkirch, France). The efficiency of gene silencing was validated through Western blot analysis. Subsequently, limiting dilution experiments were conducted on PC cells transfected with SPP1 siRNA or an NC. Spheroids were formed both in vitro and in vivo.

### Limiting dilution assay In vitro

The PC cells were cultured in DMEM/F12 (Gibco), supplemented with 20 ng/mL EGF, 20 ng/mL bFGF, and 1% FBS for 1 week after being seeded in 96-well ultra-low attachment culture plates (Corning Incorporated Life Sciences). Cell seeding densities included 2, 4, 8, 16, 32, and 64 cells per well. The ELDA software was utilized to calculate the percentage of CSCs [22]. The experimental process was repeated three times.

### Spheroid assay

The PC cells were cultured in DMEM/F12 (Gibco) supplemented with 20 ng/mL EGF, 20 ng/mL bFGF, and 1%FBS for one week after being seeded on 96-well ultra-low attachment culture plates (Corning Incorporated Life Sciences) at a density of 300 cells/well. Subsequently, a spheroid count was performed, and representative images of the samples were captured. The experiment was repeated three times.

### Flow-cytometric analysis

Primary PC patients and PC cells were treated with primary anti-CD90 or anti-CD133 antibodies (Biolegend, Inc., CA, USA) for 30 min at RT. Subsequently, the cells were subjected to flow assisted cell sorting using a Beckman Coulter MoFlo XDP cell sorter (Indianapolis, IN, USA) following the manufacturer’s directions. RT-PCR analysis was then performed on the specified cells obtained from three independent tests.

### Apoptosis assay

The PC cells overexpressing lncRNA FOXD1-AS1 and the control PC cells were treated with 5-FU at a concentration of 10 μM for 48 h. Subsequently, the cells were stained with 7-amino actinomycin D (7-AAD) and Annexin V for 15 min at RT in the dark. Apoptotic cells were identified using a flow cytometer and the Annexin VFITC Apoptosis Detection Kit I (BD Pharmingen, CA, USA) in accordance with the manufacturer's instructions.

### Animal models

Animal experiments adhered to the guidelines established by the animal care and use committees of the Third Affiliated Hospital of Naval Medical University (Shanghai, China). The experimental animals used in this study were 4–6-week-old NOD-SCID mice obtained from SIPPR-BK Experimental Animal Co. in China. These mice were housed in a controlled environment meeting pathogen-free standards and provided with appropriate nutrition. For the in vivo limiting dilution assay, PC cells were serially diluted to the required concentrations (1 × 10^3^, 5 × 10^3^, 1 × 10^4^, and 5 × 10^4^) and then mixed with 100 μl Matrigel (1:1). Subsequently, the hybridized cells were administered via subcutaneous injection into NOD-SCID mice, with a total of five animals per group, assigned in a randomized manner. After 2 months, the mice were euthanized, and the quantity of tumors was enumerated.

### Western blotting assay

The samples were collected using a cell lysis buffer and processed according to a previously reported method [23]. In this study, a total of 25 μg of proteins underwent separation using the SDS-PAGE technique. Subsequently, the separated proteins were transferred onto an NC membrane. The NC membrane was blocked with 5% non-fat milk before the addition of the primary antibody and IRDye 800CW-conjugated secondary antibody. Fluorescence intensity was measured using the LI-COR Imaging System (LI-COR Biosciences). The primary antibodies used were SPP1 (1:1000, ab283656, Abcam), β-actin (1:1000; 27309-1-AP, Proteintech).

### RNA immunoprecipitation (RIP) assay

RIP experiments were carried out using the Magna RIP RNA-Binding Protein Immunoprecipitation Kit (#17-701, Millipore, Bedford, MA, USA) [23].

### Luciferase reporter assay

Segments of FOXD1-AS1, encompassing both the mutant (MUT) and wild-type (WT) miR-570-3p binding sites, were synthesized and provided by Shanghai GenePharma. Subsequently, luciferase reporter assays were conducted following previously established protocols [23].

### RNA-sequencing

The total RNA extracted from the tissues was purified using the RNeasy Mini kit (Qiagen GmbH). The RNA integrity number (RIN) was determined using the Agilent Bioanalyzer 2100 equipment manufactured by Agilent Technologies, Inc. to assess RNA integrity. The RNA clean-up process utilized the RNA Clean XP kit from Beckman Coulter, Inc. Subsequently, DNA residues were eliminated with the RNase-free DNase Set from Qiagen GmbH. RNA quality and concentration were evaluated using the NanoDrop 2000 instrument from Thermo Fisher Scientific, Inc. Ribosomal RNA was depleted using the NEBNext rRNA Depletion kit from New England BioLabs, Inc. Following this step, 1 µg of total RNA was employed for library preparation using the VAHTSTM mRNA-seq v2 library Prep kit (Vazyme Biotech Co., Ltd.), following the manufacturer’s instructions. The RNA strands were fragmented and converted into double-stranded complementary DNA (cDNA). Adapter ligation was performed as the last step in the refinement process. The ligated cDNA underwent PCR amplification (Illumina, Inc.) for a total of 15 cycles. Each cycle included denaturation at 98 °C for 10 s, annealing at 60 °C for 30 s, and extension at 72 °C for 30 s. This amplification aimed to generate a library of sufficient size for subsequent sequencing, and DNA polymerase I (New England BioLabs, Inc.) was utilized for universal PCR amplification. The quality of the library was assessed using the Agilent Bioanalyzer 2100, and RNA-seq was carried out on an Illumina Hiseq 4000 instrument from Illumina, Inc. The SOAP tool was employed to compute the expression levels of lncRNAs and mRNAs. R software version 3.1 was used to identify differentially expressed lncRNAs and mRNAs based on the criteria of fold-change > 8 and a false discovery rate (FDR) ≤ 0.01.

### Statistical analysis

The experiments were repeated a minimum of three times and the data are reported in the form of mean ± SD. Statistical analyses were carried out using GraphPad Prism (GraphPad, USA). T-tests or Bonferroni Multiple Comparisons Tests were employed for statistical analysis, with a significance level set at *p < 0.05,NS p > 0.05. A p-value below the threshold of 0.05 was considered statistically significant.

## Results

### LncRNA FOXD1-AS1 is up-regulated in pancreatic cancer CSCs.

**Fig. 1:**
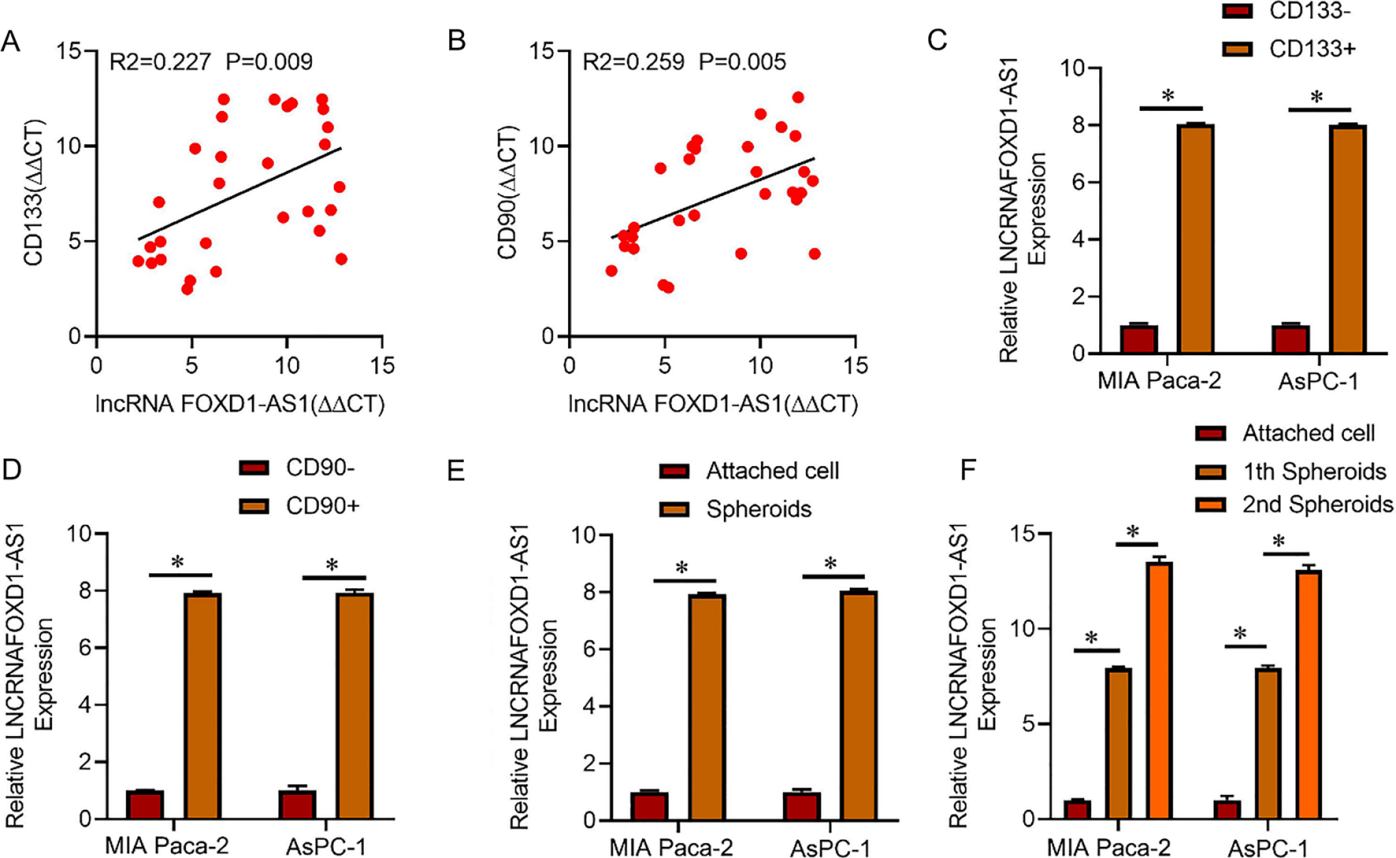
lncRNA FOXD1-AS1 expression is up-regulated in pancreatic cancer CSCs. **A** Spearman’s correlation analysis was performed on RT-PCR data to investigate the relationship between lncRNA FOXD1-AS1 concentration and CD133 in primary PC cells (n = 30). The data, transformed to β-actin as △Ct, were analyzed to establish a correlation. **B** The association between lncRNA FOXD1-AS1 levels and CD90 in primary PC cells (n = 30) was examined using RT-PCR, and the data were normalized to β-actin as △Ct. Spearman’s correlation analysis was employed for statistical evaluation. **C** Real-time PCR was utilized to assess lncRNA FOXD1-AS1 expression in CD133 + and CD133- PC cells. **D** RT-PCR was employed to compare lncRNA FOXD1-AS1 expression in CD90 + and CD90- PC cells. **E** The expression of lncRNA FOXD1-AS1 in PC spheroids and adherent cells was evaluated through real-time PCR. **F** Serial passages of PC spheroids were subjected to RT-PCR to evaluate the expression of lncRNA FOXD1-AS1

Evidence supports CD133 and CD90 as well-established CSC markers [24]. As depicted in Fig. 1A, B, Pearson correlation analysis revealed a positive association between the levels of lncRNA FOXD1-AS1 and the expression of CD133 and CD90 in tumor cells obtained from primary PC tissues. Subsequently, we employed flow cytometry sorting or sphere development techniques to enrich the population of pancreatic cancer CSCs. Consistent with the anticipated outcomes, the expression levels of lncRNA FOXD1-AS1 were significantly elevated in isolated CD133 + or CD90 + PC cells compared to CD133- or CD90- PC cells (Fig. 1C, D). Moreover, lncRNA FOXD1-AS1 expression was dramatically increased in PC spheres compared to adherent PC cells (Fig. 1E). Notably, lncRNA FOXD1-AS1 expression showed a gradual increase in serial passages of PC spheroids (Fig. 1F).

### lncRNA FOXD1-AS1 promoted carcinogenesis and self-renewal in pancreatic cancer CSCs

**Fig. 2:**
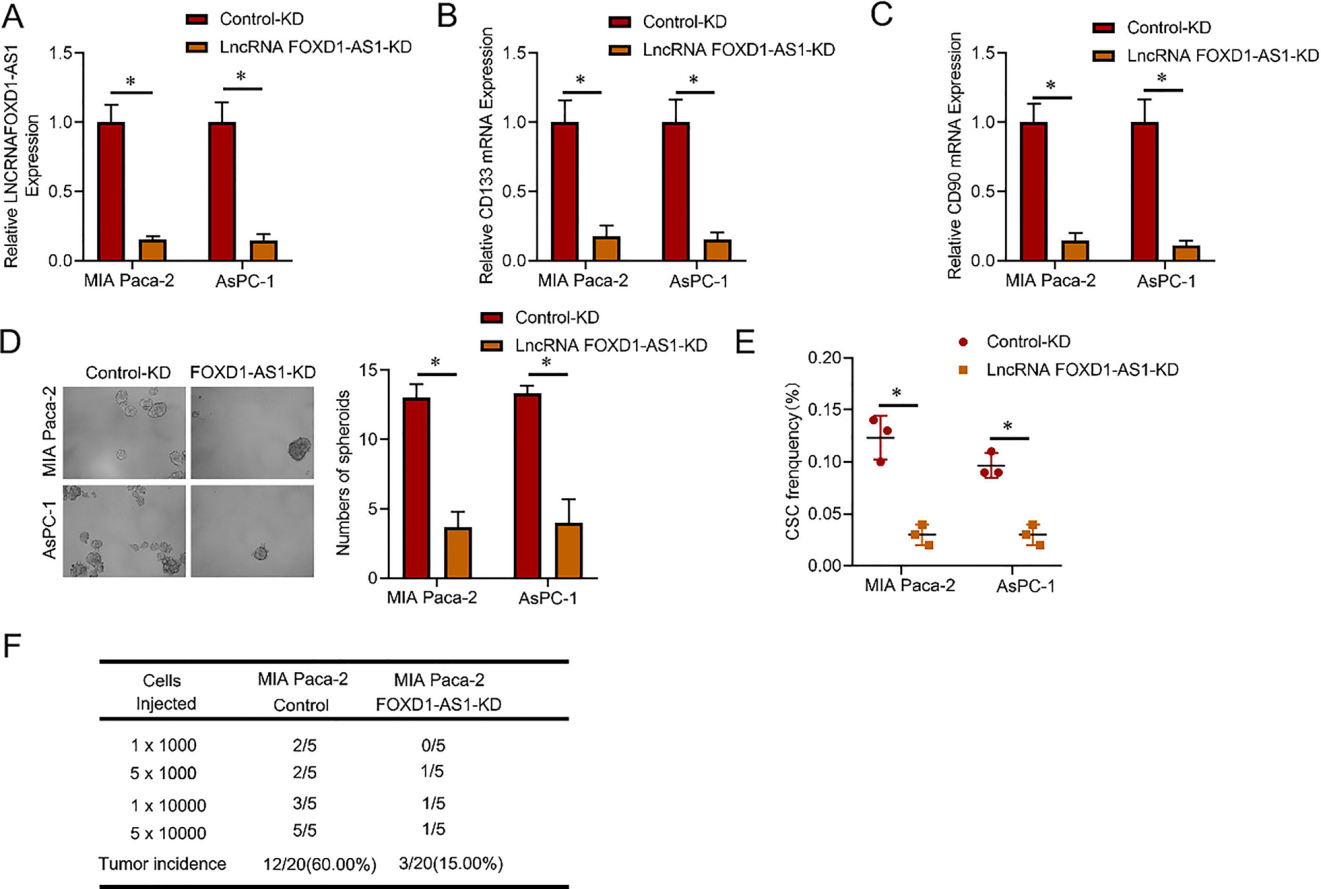
lncRNA FOXD1-AS1 knockdown inhibited pancreatic cancer CSC self-renewal and tumorigenesis. **A** MIA Paca-2 and AsPC-1 cells were infected with lncRNA FOXD1-AS1 knockdown virus, and the overexpression effect was verified by real-time PCR. **B** and **C** RT-PCR analysis was conducted to assess the expression levels of CD133 and CD90 in lncRNA FOXD1-AS1 knockdown and control PC cells.** D** The study utilized a spheroid formation experiment to investigate the effects of lncRNA FOXD1-AS1 knockdown on cellular behavior in PC cells, in contrast to control cells. Representative visuals depict the formation of spheres. **E** The percentage of pancreatic cancer CSCs in lncRNA FOXD1-AS1 knockdown cells and control PC cells was compared using an in vitro limiting dilution assay. **F** An in vivo limiting dilution assay was performed on lncRNA FOXD1-AS1 knockdown cells and control PC cells. Tumors were monitored over 2 months, with n = 5 for each group

**Fig. 3:**
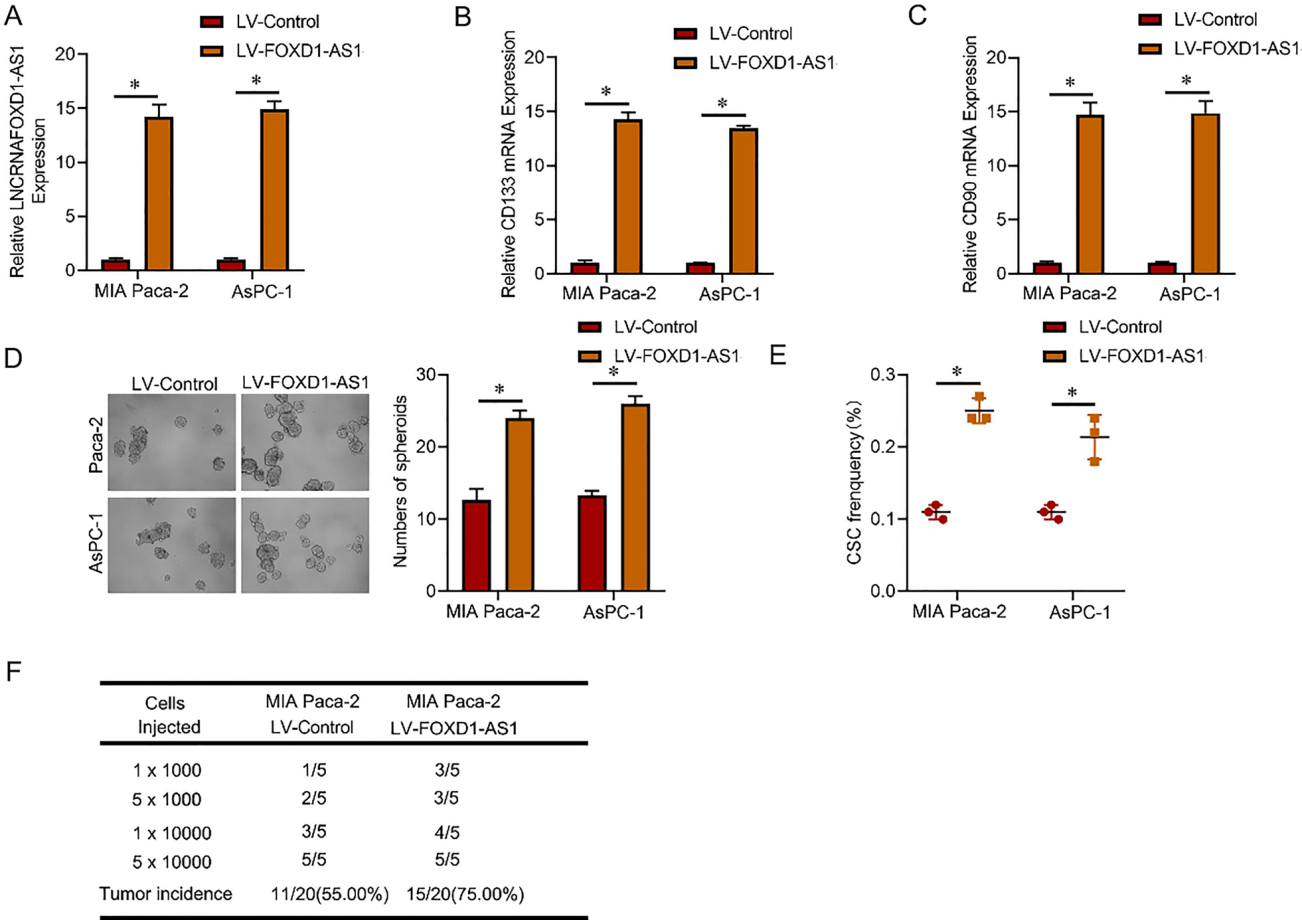
lncRNA FOXD1-AS1 overexpression promotes pancreatic cancer CSC self-renewal and tumorigenesis. **A** MIA Paca-2 and AsPC-1 cells were infected with a virus overexpressing lncRNA FOXD1-AS1, and the efficacy of overexpression was subsequently assessed using RT-PCR. **B** and **C** RT-PCR analysis was employed to evaluate CD133 and CD90 expression in control PC cells and lncRNA FOXD1-AS1 overexpressing cells. **D** The study utilized a spheroid formation assay to examine the impact of lncRNA FOXD1-AS1 overexpression on cellular behavior in PC cells, with representative images of spheres shown.** E** The percentage of pancreatic cancer CSCs in lncRNA FOXD1-AS1 overexpressing and control PC cells, compared using an in vitro limiting dilution assay. **F** An in vivo limiting dilution assay was conducted on lncRNA FOXD1-AS1 overexpressing and control PC cells. Tumors were monitored over 2 months with n = 5 for each group

MIA Paca-2 and AsPC-1 cells were subjected to infection with a lncRNA FOXD1-AS1 knockdown virus to explore the potential biological significance of this RNA in pancreatic cancer CSCs. The knockdown efficiency was subsequently assessed using real-time PCR (Fig. 2A). Compared to the control PC cells, lncRNA FOXD1-AS1 knockdown cells exhibited decreased expression of CSC markers (Fig. 2B C). Additionally, lncRNA FOXD1-AS1 knockdown cells demonstrated a reduced ability for self-renewal (Fig. 2D). The fraction of CSCs in lncRNA FOXD1-AS1 knockdown PC cells was significantly diminished, as evidenced by an in vitro limiting dilution experiment (Fig. 2E). Moreover, the in vivo limiting assay indicated a substantial reduction in the tumorigenic capacity of hepatoma cells upon lncRNA FOXD1-AS1 knockdown (Fig. 2F). Subsequently, MIA Paca-2 and AsPC-1 cells were infected with a virus carrying the overexpression of lncRNA FOXD1-AS1. The degree of overexpression was assessed using real-time PCR (Fig. 3A). lncRNA FOXD1-AS1 overexpressing cells displayed elevated expression of CSC markers compared to control PC cells (Fig. 3B, C). Furthermore, lncRNA FOXD1-AS1 overexpressing cells exhibited an enhanced capacity for self-renewal (Fig. 3D). The percentage of CSCs in lncRNA FOXD1-AS1 overexpressing PC cells was significantly higher, as indicated by an in vitro limiting dilution experiment (Fig. 3E). Additionally, the in vivo limiting investigation demonstrated that the overexpression of lncRNA FOXD1-AS1 in PC cells significantly increased their carcinogenic potential (Fig. 3F). These findings collectively suggest that lncRNA FOXD1-AS1 promotes carcinogenesis and CSC self-renewal in pancreatic cancer.

### LncRNA FOXD1-AS1 enhanced SPP1 expression by acting as a competing endogenous RNA (ceRNA) for miR-570-3p

**Fig. 4:**
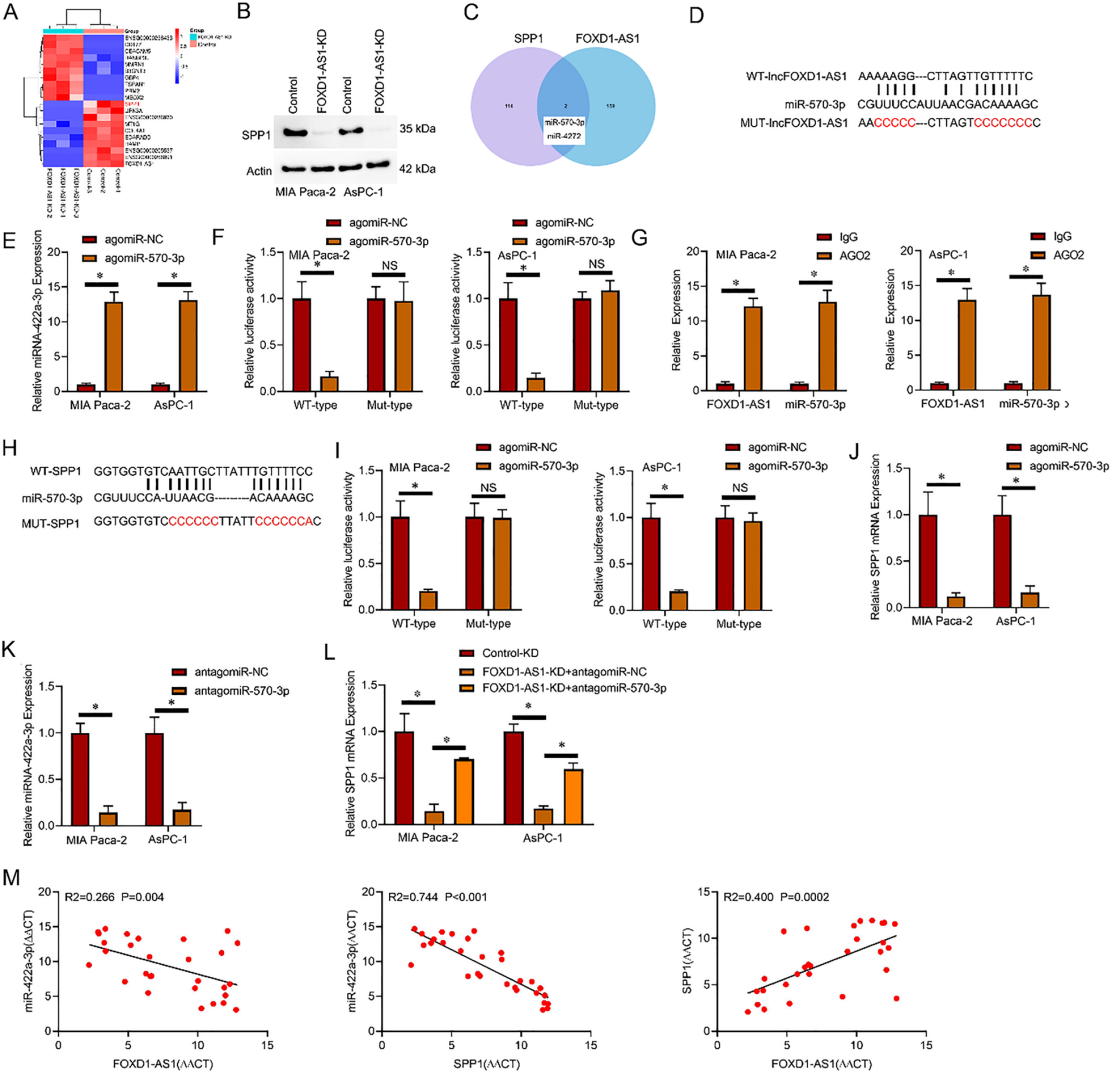
lncRNA FOXD1-AS1 enhanced SPP1 expression by sponging miR-570-3p as a ceRNA. **A** Heatmap illustrating differentially expressed genes in control and lncRNA FOXD1-AS1-KD PC cells. **B** Western blot analysis of SPP1 protein in lncRNA FOXD1-AS1 knockdown and PC control cells. **C** Venn diagram demonstrating the common complementary binding sites shared by miR-570-3p and miR-4272 in lncRNA FOXD1-AS1 and SPP1. **D** Bioinformatics analysis identified specific regions where miR-570-3p interacts with lncRNA FOXD1-AS1. **E** RT-PCR quantifying miR-570-3p expression. **F** Luciferase reporter assays conducted in specified PC cell lines. **G** The enrichment of lncRNA FOXD1-AS1 and microRNA miR-570-3p in Ago2-immunoprecipitates compared to the IgG control. **H** Bioinformatics analysis identified specific regions within the SPP1 gene targeted by miR-570-3p. **I** Luciferase reporter assays conducted on specified PC cell lines. **J** RT-qPCR analysis of SPP1 mRNA levels in the indicated PC cell lines. **K** Detection of miR-570-3p expression in PC cells by RT-qPCR following transfection with antagomir-NC or antagomiR-570-3p. **L** SPP1 mRNA levels in the PC cell lines were quantified through RT-qPCR. **M** RT-PCR was conducted to examine the relationship between the concentrations of lncRNA FOXD1-AS1, miR-570-3p, and SPP1 in primary PC cells (n = 30). The data were normalized using β-actin as △Ct and afterward analyzed using Spearman's correlation method

To elucidate the mechanism by which lncRNA FOXD1-AS1 promotes pancreatic cancer CSC expansion, we conducted RNA sequencing to assess the gene expression profile in lncRNA FOXD1-AS1-KD and control PC cells. The transcriptomic analysis revealed that 275 genes exhibited decreased expression (> eightfold, P < 0.01), and among the top 10 downregulated genes was SPP1, which has been reported to regulate CSCs [25] (Fig. 4A). Furthermore, SPP1 expression was reduced in lncRNA FOXD1-AS1 knockdown cells (Fig. 4B). Based on these findings, it was hypothesized that lncRNA FOXD1-AS1 functions as a competing endogenous RNA (ceRNA) for a microRNA that would otherwise regulate the expression of SPP1. In support of this role, a subcellular fractionation study revealed that lncRNA FOXD1-AS1 exhibited preferential localization within the cytoplasm of PC cells (Additional file 1: Fig. S1). The identification of potential miRNAs with complementary binding sites in both lncRNA FOXD1-AS1 and SPP1 led to the identification of miR-570-3p and miR-4272 as promising candidates (Fig. 4C and Additional file 1: Figs. S2, S3A). To further investigate, we performed co-transfections of agomiR-NC or agomiR-570-3p along with MUT-LncRNA FOXD1-AS1 or WT-LncRNA FOXD1-AS1 variants into PC cells, confirming the effects using luciferase reporter assays. The luciferase activity of the WT-LncRNA FOXD1-AS1 constructs in PC cells decreased when exposed to agomiR-570-3p, as depicted in Fig. 4D–F. However, the activity of the MUT-LncRNA FOXD1-AS1 construct remained unaffected by agomiR-570-3p. Importantly, the introduction of agomiR-570-3p did not reduce luciferase activity in PC cells when either the WT lncRNA FOXD1-AS1 or MUT lncRNA FOXD1-AS1 construct was utilized (Additional file 1: Fig. S3B, C). Furthermore, RIP assays revealed that lncRNA FOXD1-AS1 and miR-570-3p were significantly more enriched in Ago2-immunoprecipitates than the IgG control (Fig. 4G). To establish the direct targeting relationship between miR-570-3p and SPP1, the complete 3'-untranslated region (3'-UTR) of the SPP1 gene was inserted downstream of the Renilla luciferase gene through cloning (Fig. 4H). Consistent with the expected outcomes, the expression of agomiR-570-3p resulted in a reduction in luciferase activity specifically in PC cells when the WT-SPP1 construct was used, while the MUT-SPP1 construct remained unaffected (Fig. 4I). Additionally, agomiR-570-3p decreased the mRNA levels of SPP1 in PC cells (Fig. 4J). The downregulated levels of SPP1 mRNA were observed to be reversed by the introduction of antagomiR-570-3p into PC cells with suppression of lncRNA FOXD1-AS1, as depicted in Figs. 4K, L. Furthermore, Pearson correlation analysis demonstrated a positive relationship between the levels of lncRNA FOXD1-AS1 and the expression of SPP1 in tumor cells derived from primary PC tissues. Conversely, miR422a-3p exhibited an inverse relationship with the expression of SPP1 and FOXD1-AS1 in tumor cells isolated from primary PC tissues (Fig. 4M). These observations suggest that lncRNA FOXD1-AS1 acts as a ceRNA by sequestering miR-570-3p, thereby enhancing the expression of SPP1.

### LncRNA FOXD1-AS1 enhanced pancreatic cancer CSCs tumorigenesis and self-renewal by targeting SPP1

To further validate the functional connection between lncRNA FOXD1-AS1 and SPP1, specific SPP1 siRNA was transfected into lncRNA FOXD1-AS1 knockdown and control PC cells. As anticipated, the administration of specific SPP1 siRNA effectively mitigated the disparity in self-renewal capacity observed between lncRNA FOXD1-AS1 overexpressing and control PC cells (Additional file 1: Fig. S4A). Furthermore, applying specific SPP1 siRNA eliminated the observed disparity in the proportion of CSCs between lncRNA FOXD1-AS1 overexpressing and control PC cells (Additional file 1: Fig. S4B). Notably, the administration of specific SPP1 siRNA effectively nullified the observed disparity in tumorigenic potential between lncRNA FOXD1-AS1 overexpressing cells and the control population of PC cells (Additional file 1: Fig. S4C). Collectively, our findings indicate that the presence of SPP1 is essential for facilitating pancreatic cancer CSC development by lncRNA FOXD1-AS1.

### LncRNA FOXD1-AS1 determines the 5-FU response in PC cells

**Fig. 5:**
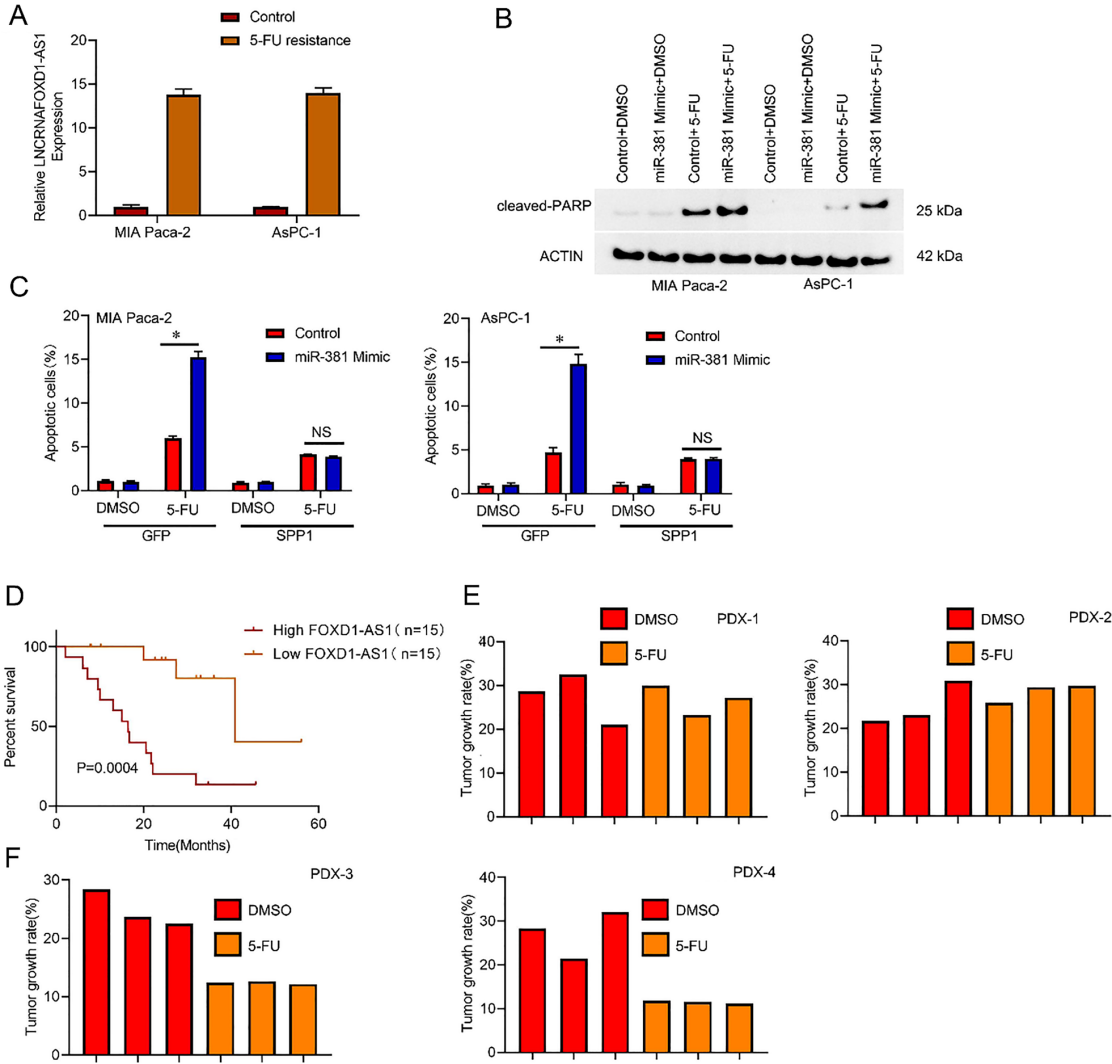
lncRNA FOXD1-AS1 determines the response to 5-FU in PC cells. **A** Real-time PCR analysis of lncRNA FOXD1-AS1 5-FU-resistant and control PC cells. **B** PC cells with lncRNA FOXD1-AS1 knockdown or control were infected with SPP1 overexpression or a negative control virus for 48 h before being treated with 5-FU (10 μM). Flow cytometry was utilized to determine the proportion of apoptotic cells. **C** The Kaplan–Meier assay was employed to determine the overall survival of PC patients treated with 5-FU in the high lncRNA FOXD1-AS1 (n = 15) or low lncRNA FOXD1-AS1 (n = 15) groups. **D** PDXs with low lncRNA FOXD1-AS1 levels generated from primary PCs were treated for 24 days with 5-FU or saline (n = 3). The growth rate of the xenograft was determined. **E** Three groups of PDXs were included in this study. PDXs were derived from primary PCs exhibiting high levels of lncRNA FOXD1-AS1. These PDXs were then treated with either 5-FU or saline for 24 days. Each group consisted of three PDXs, and the rate of xenograft growth was determined

Research suggests the involvement of CSCs in modulating chemoresistance [26]. A notable elevation in lncRNA FOXD1-AS1 expression levels was observed in PC cell lines exhibiting resistance to 5-FU (Fig. 5A). Moreover, the introduction of SPP1 reversed the sensitivity of lncRNA FOXD1-AS1 knockdown PC cells to 5-FU-induced cell apoptosis (Fig. 5B). Importantly, Kaplan–Meier analysis of PC patients who received 5-FU treatment after surgery revealed that low lncRNA FOXD1-AS1 expression was associated with longer survival time after 5-FU therapy (Fig. 5C). Furthermore, the patient-derived xenograft (PDX) model showed that lncRNA FOXD1-AS1 high PC tissues were resistant to 5-FU treatment (Fig. 5D), while lncRNA FOXD1-AS1 low PC tissues were sensitive to 5-FU treatment (Fig. 5E). These findings suggest that lncRNA FOXD1-AS1 may serve as an effective indicator for 5-FU treatment in PC.

### FOXD1-AS1 up-regulation in PC is mediated by m^6^A RNA methylation

**Fig. 6:**
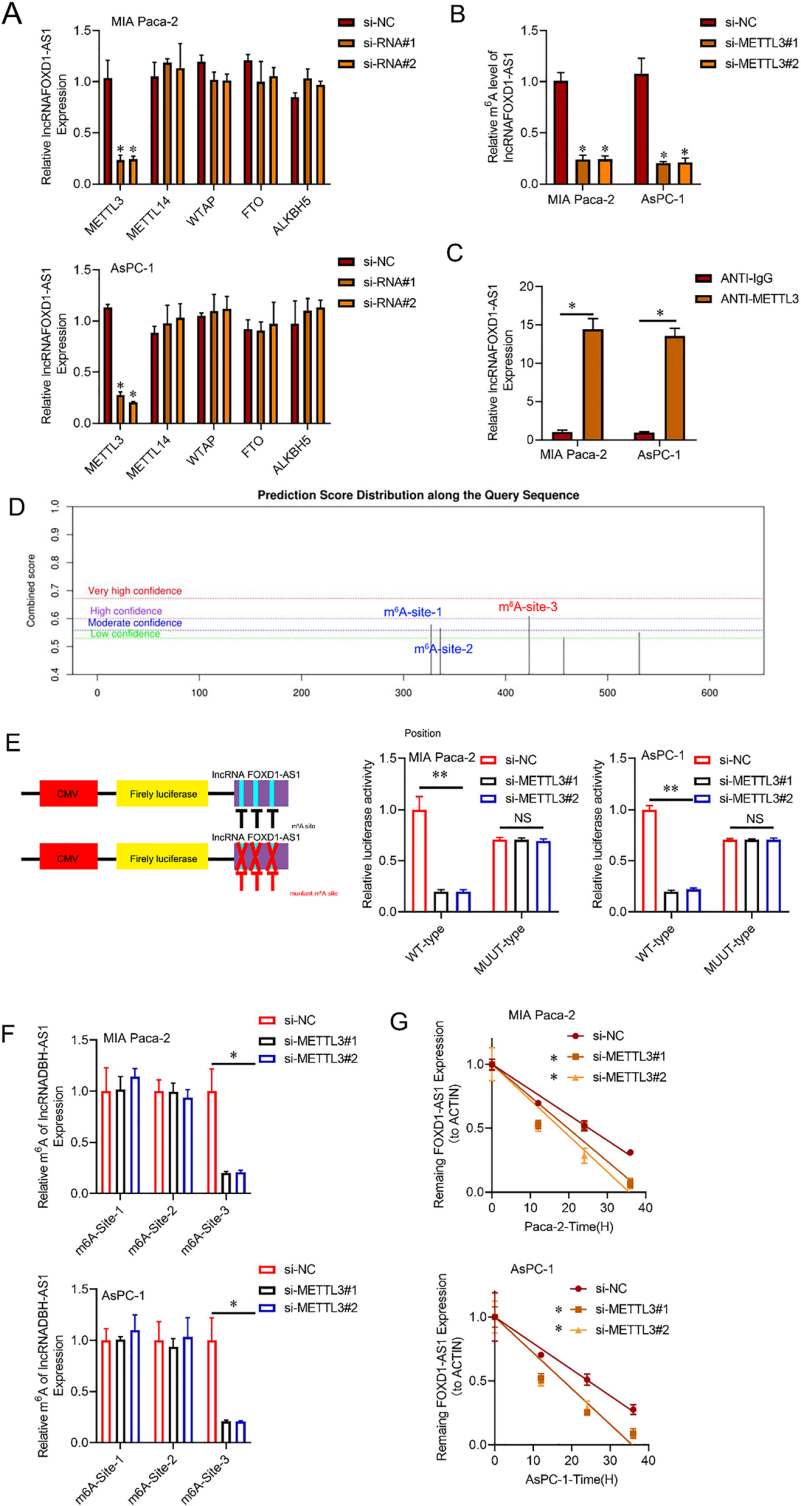
m6A RNA methylation underlies the upregulation of FOXD1-AS1 in PC. **A** qRT-PCR was used to analyze the expression of lncRNA FOXD1-AS1 in the specified PC cell lines. **B** m^6^A levels in lncRNA-FOXD1-AS1 cells were determined using m6A-qPCR in PC cells transfected with si-NC or si-METTL3 #1, #2. **C** A RIP assay with anti-METTL3 antibodies was conducted to assess the interaction between METTL3 and lncRNA-FOXD1-AS1. **D** The SRAMP website was utilized to identify the specific m6A methylation locations of lncRNA-FOXD1-AS1. **E** In PC cells co-transfected with siMETTL3#1, #2, or siNC, the relative luciferase activity of pMIR-REPORT-LNCRNA-DBH-AS1 with either WT or MUT (A-to-T mutation) m^6^A sites was measured. Renilla luciferase activity was used as the standard to assess and normalize Firefly luciferase activity. **F** m^6^A-qPCR was employed to determine the m^6^-site levels in lncRNA-DBH-AS1 in PC cells transfected with siMETTL3#1, #2, or siNC. **G** Relative luciferase activity of pMIR-REPORT-lncRNA-FOXD1-AS1 co-transfected with siMETTL3#1, #2, or siNC in MIA Paca-2 and AsPC-1 cells with WT or MUT (A-to-T mutation) m6A sites, respectively. Renilla luciferase activity was used as the standard to assess and normalize Firefly luciferase activity. **F** lncRNA-FOXD1-AS1 levels in PC cells treated with actinomycin D (10 μM) were compared to ACTIN using qRT-PCR

We conducted additional investigations to delve into the fundamental regulatory mechanisms through which lncRNA-FOXD1-AS1 was up-regulated in PC. Subsequently, we administered DNA methylation (5-AZA) or histone deacetylase (HDAC) inhibitors (TSA) to PC cell lines. According to the findings presented in Additional file 1: Fig. S5A, it can be inferred that the expression of lncRNA-FOXD1-AS1 is unaffected by histone acetylation or DNA methylation. Furthermore, the lncRNA-FOXD1-AS1 expression in PC cells was not significantly affected by RNA interference to inhibit Dicer, the enzyme regulating microRNA processing (Additional file 1: Fig. S5B). This led us to shift our focus towards examining RNA methylation, a pervasive regulatory mechanism that has surfaced to govern gene expression. A comparative analysis was conducted using siRNA-mediated knockdown to assess the impact of different m^6^A components. These analyses revealed a significant reduction in lncRNA-FOXD1-AS1 expression upon inactivation of the METTL3 methyltransferase, as compared to WTAP or METTL14 depletion. Furthermore, the depletion of the m^6^A demethylases ALKBH5 or FTO did not substantially affect the lncRNA-FOXD1-AS1 expression (Fig. 6A and Additional file 1: Fig. S5C). To further validate m^6^A hyper-methylation mediated by METTL3 in PC cell lines, with a specific focus on lncRNA-FOXD1-AS1, we employed m6A-qPCR. Additionally, RNA immunoprecipitation experiments provided evidence supporting the interaction and binding between METTL3 and lncRNA-FOXD1-AS1 mRNA in PC cells (Fig. 6B, C). Utilizing the SRAMP database to investigate the lncRNAFOXD1-AS1 sequence, we identified three potential m^6^A methylation sites (Fig. 6D). Assessments using luciferase reporters and mutagenesis were conducted to thoroughly explore the influence of m6A mutation on lncRNA-FOXD1-AS1. Luciferase reporter assays and mutagenesis were subsequently employed to comprehensively understand the impact of m6A mutations on lncRNA-FOXD1-AS1. As anticipated, METTL3 knockdown resulted in reduced luciferase activity, specifically in the wild-type group, while the mutant group remained unaffected. These results underscore the significance of m^6^A modification in lncRNA-FOXD1-AS1. Moreover, METTL3 was observed to induce hyper-methylation at m^6^A-site3 of lncRNA-FOXD1-AS1 in PC cell lines (Fig. 6E, F). Our findings also confirm that the suppression of METTL3 leads to reduced stability of lncRNA-FOXD1-AS1 mRNA in pancreatic cancer cells. This provides compelling evidence that METTL3 promotes m6A hypermethylation of lncRNA-FOXD1-AS1, thereby enhancing its mRNA stability in PC (Fig. 6G).

### YTHDF1 improves lncRNA-FOXD1-AS1 mRNA stability in a m^6^A-dependent manner

**Fig. 7:**
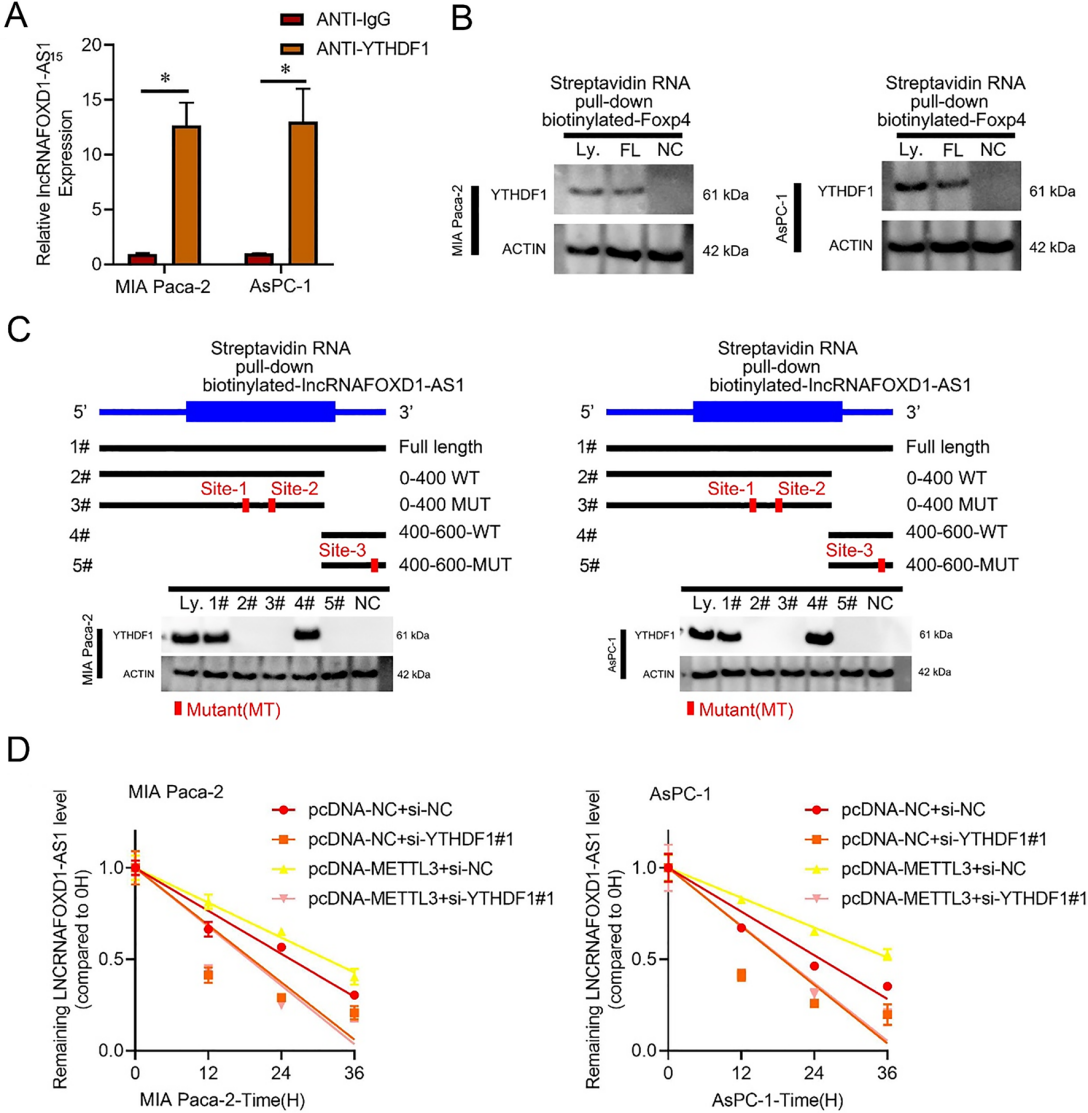
YTHDF1 improves lncRNA-FOXD1-AS1 mRNA stability in a m^6^A-dependent manner. **A** The binding of lncRNA-FOXD1-AS1 to the YTHDF1 protein in PC was confirmed by RIP-qPCR.** B** YTHDF1 was assessed via Western blotting in PC using cell lysate, full-length biotinylated lncRNA-FOXD1-AS1, and only beads (NC). **C** Western blot analysis of YTHDF1 in PC with cell lysate (Ly.), completely biotinylated-lncRNA-FOXD1-AS1 (#1), lncRNA-FOXD1-AS1(0–400) with m^6^A pattern site 1 and 2-WT (#2) and site 1 and 2-MUT (#3), lncRNA-FOXD1-AS1(400–600) with m^6^A pattern site 3-WT (#4) and site 3-MUT (#5), and beads alone (NC). **D** Analysis of lncRNA-FOXD1-AS1 levels with ACTIN using qRT-PCR in PC cells treated with actinomycin D (10 μM)

**Fig. 8:**
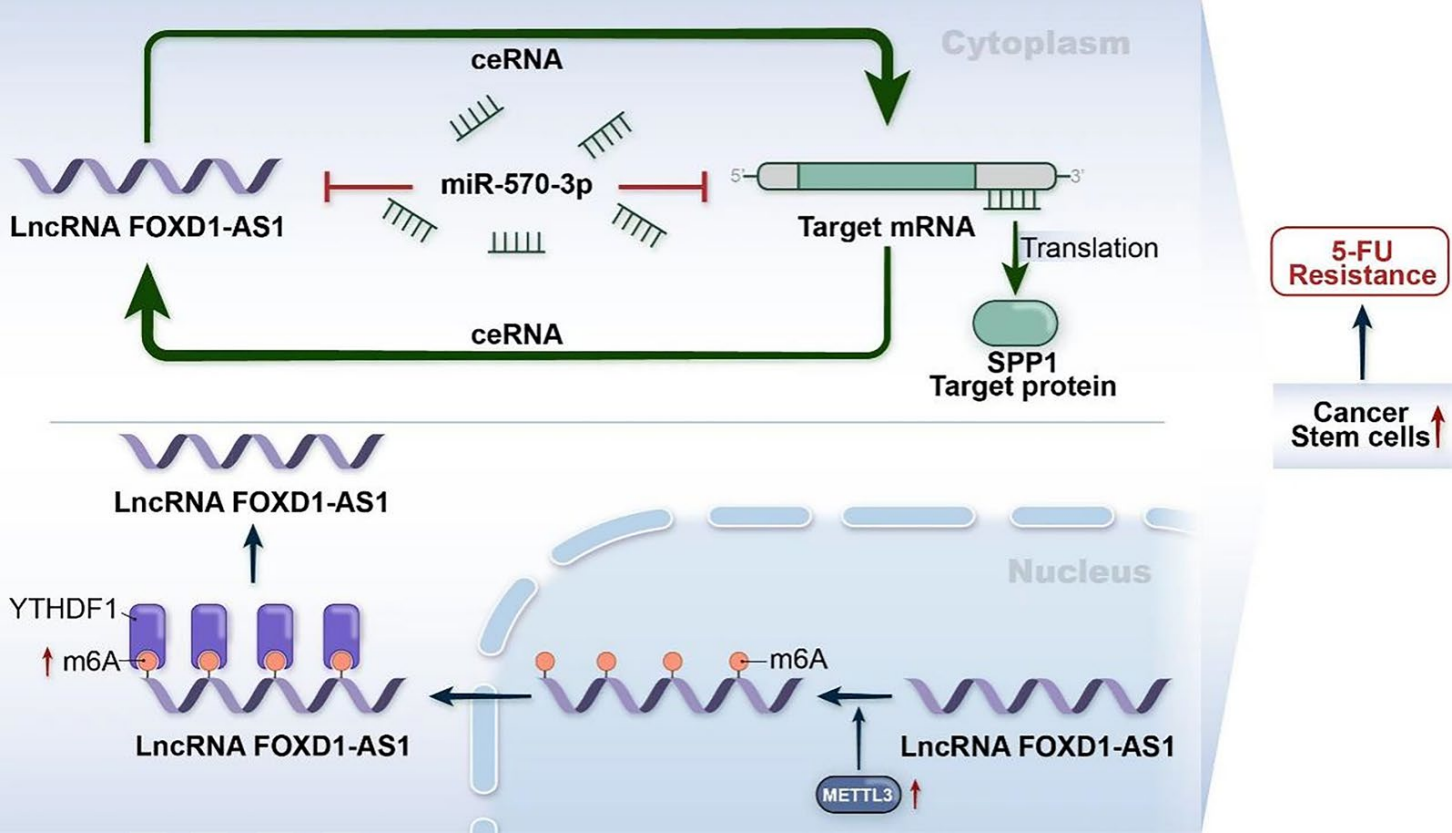
A functional model illustrating the mechanism by which lncRNA-FOXD1-AS1 enhances pancreatic cancer stem cell properties and contributes to 5-FU resistance

According to the literature, the stability and translation of mRNA, regulated by "reader proteins", are influenced by RNA m^6^A modifications [27]. Our analysis unveiled that interference with YTHDF1, as opposed to IGF2BP1, IGF2BP2, IGF2BP3, YTHDF2, or YTHDF3, significantly reduced lncRNA-FOXD1-AS1 mRNA levels in PC cells (Additional file 1: Fig. S6A, B). RNA immunoprecipitation experiments provided evidence of YTHDF1 binding and recognition of lncRNA-FOXD1-AS1 mRNA in PC cells (Fig. 7A). Streptavidin RNA pull-down assays further revealed that in PC cells, YTHDF1 selectively bound to the full-length transcripts of lncRNA-FOXD1-AS1 (Fig. 7B). Notably, the RNA pull-down experiments confirmed that in PC cells, YTHDF1 predominantly associated with the lncRNA-FOXD1-AS1 m^6^A-site3 rather than m^6^A-site-1 or-2 (Fig. 7C and Additional file 1: Fig. S6C). Additionally, our findings demonstrated that the extended half-life of lncRNA-FOXD1-AS1transcripts in PC cells with up-regulated METTL3 countered the prolonged half-lives induced by YTHDF1 knockdown (Fig. 7D and Additional file 1: Fig. S6D). These results suggest direct recognition of methylated lncRNA-FOXD1-AS1 transcripts by the m^6^A "reader" YTHDF1. This recognition preserves the stability of the transcripts, preventing degradation and naturally enhancing their production through an m^6^A-YTHDF1-dependent process (Fig. 8).

## Discussion

The current study observed an upregulation in the expression of lncRNA FOXD1-AS1 in primary pancreatic cancer stem cells (CSCs). Functioning as a key oncogenic driver, lncRNA FOXD1-AS1 plays a crucial role in the self-renewal and tumorigenesis of pancreatic cancer CSCs. Mechanistic exploration revealed a novel regulatory axis involving lncRNA FOXD1-AS1, miR-570-3p, and SPP1 in pancreatic cancer CSCs. Notably, lncRNA FOXD1-AS1 emerged as a key determinant in the responsiveness of PC cells to 5-FU treatment. Furthermore, the inhibition of lncRNA FOXD1-AS1 effectively reversed resistance to 5-FU. Analysis of the patient cohort further suggested that low levels of lncRNA FOXD1-AS1 could potentially serve as a predictive marker for 5-FU clinical benefit in PC patients.

Accumulating evidence underscores the crucial role of lncRNAs in regulating CSCs or T-ICs. Both the loss and gain of lncRNA functions contribute to the self-renewal and carcinogenesis of CSCs or T-ICs [28–31]. For instance, lncRNA SNHG5 has been found to enhance cancer stem cell-like features and HCC proliferation by modulating UPF1 and the Wnt-signaling pathway [32]. Similarly, the long non-coding RNA KLK8 has been implicated in influencing stem cell properties in colon cancer [33]. However, the precise functional role of lncRNA FOXD1-AS1 in pancreatic cancer CSCs remains undetermined. Our study reveals that pancreatic cancer CSCs exhibit elevated lncRNA FOXD1-AS1 compared to normal cells. Forced expression of lncRNA FOXD1-AS1 resulted in an increase in CSC markers and stemness-associated transcription factors. CSCs, known for their tumor-initiating and self-renewal capabilities, displayed reduced potential for tumorigenicity and self-renewal upon lncRNA FOXD1-AS1 knockdown. Collectively, these findings underscore the significant role of lncRNA FOXD1-AS1 in the growth of cancer CSCs in pancreatic cancer, highlighting its potential as a therapeutic target.

In our current research, we utilized a bioinformatic analysis to identify specific genes targeted by lncRNA FOXD1-AS1. Subsequent experimental validation revealed that lncRNA FOXD1-AS1 has the potential to enhance the expression of Osteopontin (OPN), also known as secreted phosphoprotein 1 (SPP1), in pancreatic cancer CSCs among the identified candidate genes. OPN/SPP1, recognized as a chemokine-like sialic acid-rich glycoprotein, has been reported to be overexpressed in various malignancies, including prostate cancer [34]. Its interaction with CD44 facilitates cell signaling, governing the motility, adhesion, and activation of neoplastic cells, ultimately contributing to the formation and metastasis of tumors [34]. Recent insights have implicated SPP1 in the regulation of CSCs or T-Ics [35]. Our study establishes that lncRNA FOXD1-AS1 up-regulates SPP1 expression by acting as a ceRNA that sponges miR-570-3p in pancreatic cancer CSCs. Notably, this investigation reveals, for the first time, the direct regulatory relationship between lncRNA FOXD1-AS1 and the gene *SPP1*. Rescue experiments further confirm that lncRNA FOXD1-AS1 contributes to the proliferation of cancer CSCs in PC, partially through the up-regulation of SPP1 expression. Beyond their role as miRNA sponges, lncRNAs exert their functions through interactions with RNA-binding proteins (RBPs), forming lncRNA-protein complexes that participate in diverse biological processes [36]. Future studies will be critical to elucidate the potential role of lncRNAFOXD1-AS1 as an RNA-binding protein (RBPs) in PC.

The chemotherapeutic agent 5-FU, known for its cytotoxic effects, serves as the cornerstone for conventional chemotherapy for PC and various other forms of cancer [37, 38]. Despite its efficacy, a significant challenge lies in the development of resistance to 5-FU in many advanced PC patients. Consequently, there is a critical need to investigate the molecular mechanisms underlying 5-FU resistance and identify reliable biomarkers for predicting the response to 5-FU. Our study reveals that PC cells with lncRNA FOXD1-AS1 depletion exhibit increased susceptibility to 5-FU-induced apoptosis and inhibition of cell growth. Additionally, Kaplan–Meier analysis of PC patients who underwent 5-FU treatment after surgery indicates that individuals with low lncRNA FOXD1-AS1 expression experience prolonged survival following 5-FU therapy. Moreover, results from the PDX model further support the notion that cases with low lncRNA FOXD1-AS1 levels benefit from 5-FU therapy. Therefore, assessing lncRNA FOXD1-AS1 expression in PC tumors before devising a treatment plan could aid in identifying patients likely to derive benefits from 5-FU therapy. This emphasizes the need for additional investigations into biomarker-guided clinical research.

Recent research has provided compelling evidence highlighting the crucial role of m^6^A modification in determining the fate of RNA molecules. This modification significantly influences various aspects of mRNA function, including translation, localization, transport, splicing, and stability [39]. In mammals, a multi-component complex comprising WTAP, METTL14, and METTL3 is responsible for mediating adenosine N6 methylation [40, 41]. Notably, our research, supported by empirical evidence demonstrating that RNA interference targeting METTL3 leads to a reduction in lncRNA-FOXD1-AS1 levels, suggests that the expression of lncRNA-FOXD1-AS1 is regulated through RNA methylation. Moreover, our findings indicate that YTHDF1 plays a pivotal role in stabilizing m6A-modified lncRNA-FOXD1-AS1 mRNA. To the best of our knowledge, this study represents the first investigation elucidating the association between m6A modification and the dysregulation of lncRNA-FOXD1-AS1 in PC.

## Conclusion

In conclusion, these data suggest that lncRNA FOXD1-AS1 may serve as a valuable predictor for assessing the efficacy of 5-FU in personalized therapy for PC This promising finding highlights the need for further research in PC and other malignancies where the lncRNA FOXD1-AS1 is known to play a functionally relevant role.

### Supplementary Information

**Additional file 1: Figure S1.** Most FOXD1-AS1 lncRNA was found in the cytoplasm of PC cells (A and B). The mean ± SDs were derived from N=3 independent observations. **Figure S2.** According to the Miranda Database, it has been observed that miR-570-3p and miR-4272 possess complementary binding sites inside the lncRNA FOXD1-AS1 and SPP1. **Figure S3.** **A** The implementation of bioinformatics analysis facilitated the identification of the specific regions where miR-4272 binds to the lncRNA FOXD1-AS1. **B** The expression study of miR-4272 was conducted through the RT-qPCR assay. **C** On the designated PC cell lines, luciferase reporter assays were performed. **Figure S4.** **A** Cells overexpressing lncRNA FOXD1-AS1 and control PC cells were transfected with siRNA targeting SPP1 or NC and subsequently underwent spheroid formation. **B** After transfecting SPP1 siRNA or negative control, lncRNA FOXD1-AS1 overexpression cells and control PC cells were put through an in vitro limiting dilution test. **C** The in vivo limiting dilution assay was performed on lncRNA FOXD1-AS1 overexpression cells and control PC cells transfected with SPP1 siRNA or NC. **Figure S5.** **B** After treating PC cells with DMSO, 5-AZA, or TSA as directed, the lncRNA FOXD1-AS1 level was measured by qPCR. **B** The level of lncRNA FOXD1-AS1 was analyzed by qPCR in pancreatic cancer cells transfected with si-Control or si-Dicer as specified. **C** qPCR was performed on pancreatic cancer cells transfected with the designated siRNA (si-Control, si-ALKBH5, si-WTAP, si-METTL14, si-METTL3, or si-FTO). **Figure S6.** **A** The PC cells were transfected with specific siRNA molecules targeting control siRNA, YTHDF-1/-2/-3, or IGF2BP-1/-2/-3. Subsequently, a qPCR assay was performed on these transfected cells. **B** The expression level of lncRNA FOXD1-AS1 in PC cells that were transfected with specific siRNAs targeting control siRNA, YTHDF-1/-2/-3, or IGF2BP-1/-2/-3 a was measured using qPCR test. **C** The m6A methylation level in PC cells by m6A-qPCR. D.qPCR analysis of lncRNA FOXD1-AS1 level in indicated PC cells. **Figure S7.** Oringal wb panel. **Table S1.** Clinicopathologic Characteristics of 30 Pancreatic Cancer Patients.

## Data Availability

The data that has been used is confidential. The data in the current study are available from the corresponding authors upon reasonable request.

## Abbreviations

lncRNA: Long noncoding RNA
CSCs: Cancer stem cells
OPN: Osteopontin
5-FU: 5-Fluorouracil
SPP1: Secreted phosphoprotein 1
T-ICs: Tumor-initiating cells
PC: Pancreatic cancer
