# Supplementary material for: LncRNA FOXD1-AS1 regulates pancreatic cancer stem cell properties and 5-FU resistance by regulating the miR-570-3p/SPP1 axis as a ceRNA
Source: Cancer Cell Int. 2024 Jan 2;24:4. doi: 10.1186/s12935-023-03181-5 (PMC10763109; doi:10.1186/s12935-023-03181-5)
Supplement: Supplementary file 1 — Additional file 1: Figure S1. Most FOXD1-AS1 lncRNA was found in the cytoplasm of PC cells (A and B). The mean ± SDs were derived from N=3 independent observations. Figure S2. According to the Miranda Database, it has been observed that miR-570-3p and miR-4272 possess complementary binding sites inside the lncRNA FOXD1-AS1 and SPP1. Figure S3. A The implementation of bioinformatics analysis facilitated the identification of the specific regions where miR-4272 binds to the lncRNA FOXD1-AS1. B The expression study of miR-4272 was conducted through the RT-qPCR assay. C On the designated PC cell lines, luciferase reporter assays were performed. Figure S4. A Cells overexpressing lncRNA FOXD1-AS1 and control PC cells were transfected with siRNA targeting SPP1 or NC and subsequently underwent spheroid formation. B After transfecting SPP1 siRNA or negative control, lncRNA FOXD1-AS1 overexpression cells and control PC cells were put through an in vitro limiting dilution test. C The in vivo limiting dilution assay was performed on lncRNA FOXD1-AS1 overexpression cells and control PC cells transfected with SPP1 siRNA or NC. Figure S5. B After treating PC cells with DMSO, 5-AZA, or TSA as directed, the lncRNA FOXD1-AS1 level was measured by qPCR. B The level of lncRNA FOXD1-AS1 was analyzed by qPCR in pancreatic cancer cells transfected with si-Control or si-Dicer as specified. C qPCR was performed on pancreatic cancer cells transfected with the designated siRNA (si-Control, si-ALKBH5, si-WTAP, si-METTL14, si-METTL3, or si-FTO). Figure S6. A The PC cells were transfected with specific siRNA molecules targeting control siRNA, YTHDF-1/-2/-3, or IGF2BP-1/-2/-3. Subsequently, a qPCR assay was performed on these transfected cells. B The expression level of lncRNA FOXD1-AS1 in PC cells that were transfected with specific siRNAs targeting control siRNA, YTHDF-1/-2/-3, or IGF2BP-1/-2/-3 a was measured using qPCR test. C The m6A methylation level in PC cells by m6A-qPCR. D.qPCR analysis [file 12935_2023_3181_MOESM1_ESM.docx]

**Additional Information**

**LncRNA FOXD1-AS1 Regulates Pancreatic Cancer Stem Cell Properties and 5-FU resistance via regulating the miR-570-3p/SPP1 axis as a ceRNA**

**Additional Materials and Methods**

**Patient-derived xenograft (PDX) model**

The process of xenografted tumor formation was observed, and the mice were killed after 10 weeks following inoculation. The primary tumor samples were acquired to create xenografts following the previously established PDX model (**1**). The xenografts, with a volume ranging from 150 to 200 mm^3^, were subsequently matched in size. The mice were later randomized to the 5-FU and Vehicle groups to administer therapeutic interventions. The mice received subcutaneous injections of 5-FU (4 mg/kg) or a control for 24 days.The sample size for each group was n=5. The measurement of tumor volumes was conducted biweekly using a caliper, employing the formula Volume=π/6*L*W2. In this equation, L indicates the longest axis of the tumor, while W denotes the shortest axis. Upon reaching a volume of around 1500 mm^3^, the mice were subjected to euthanasia using CO_2_. Subsequently, the tumors were either sectioned or frozen for further analysis. The animal procedures used in this study adhered to the recommended standards for animal care. The Ethics Committee at Changhai Hospital, located in Shanghai, China, examined and authorized the study's methodologies.

**FOXD1-AS1 degradation rate**

The FOXD1-AS1 mRNA degradation rate was evaluated in the presence of the transcription inhibitor actinomycin D using the established methodology (**2**).

**m^6^A meRIP-qPCR**

The MeRIP tests were conducted as per the guidelines provided by the manufacturer (Magna MeRIP m6A Kit).

**Real-time PCR**

The isolation of total RNA from cells or tissues was performed using TRIZOL (Invitrogen) as per the guidelines set by the manufacturer. RNA purity was quantified using a UV spectrophotometer, namely the NanoDrop ND-1000. Additionally, the validation of RNA integrity was performed by agarose gel electrophoresis. The M-MLV RTase cDNA Synthesis Kit (Promega) was used to convert the extracted RNA into cDNA. RT-PCR analysis was conducted using Roche's LightCycler 480 System and SYBR Green PCR Kit. A single cycle of 95 °C for five minutes was followed by up to 40 cycles of denaturation at 95 °C for 15 seconds, annealing at 60 °C for 30 seconds, and extension at 72 °C for 30 seconds. Melting curves obtained after the experiment verified the specificity of the primers.

**
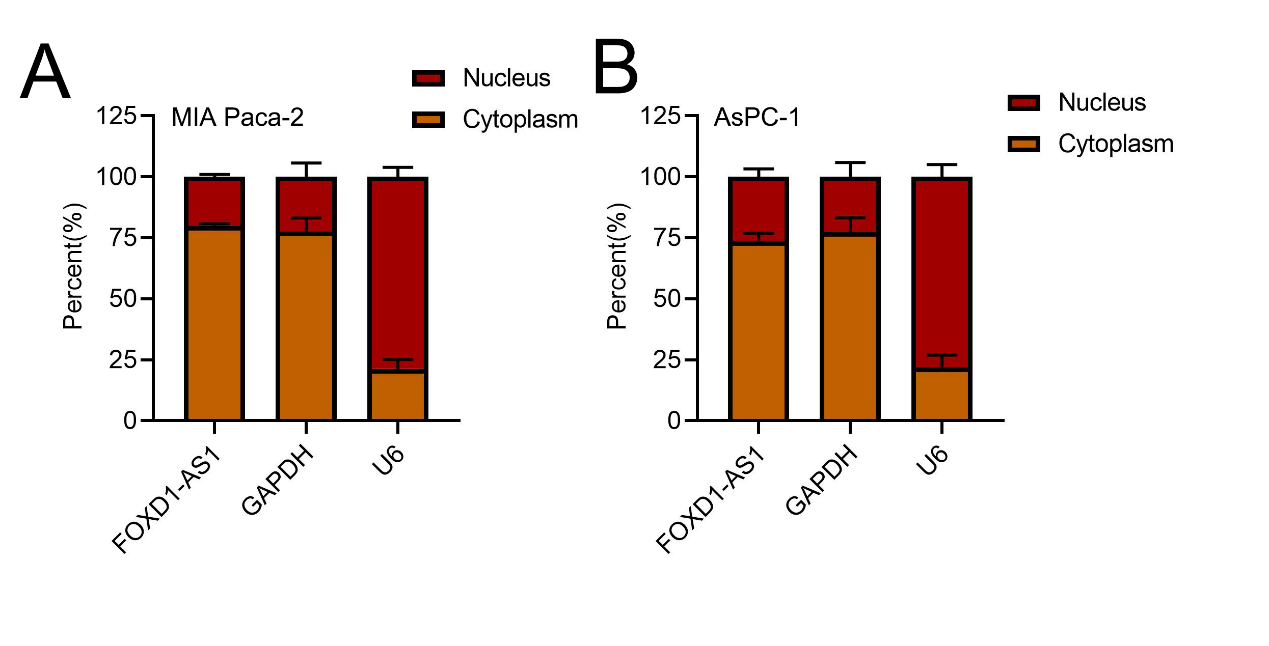
Figure S1:**  Most FOXD1-AS1 lncRNA was found in the cytoplasm of PC cells (**A** and **B**). The mean ± SDs were derived from N=3 independent observations.


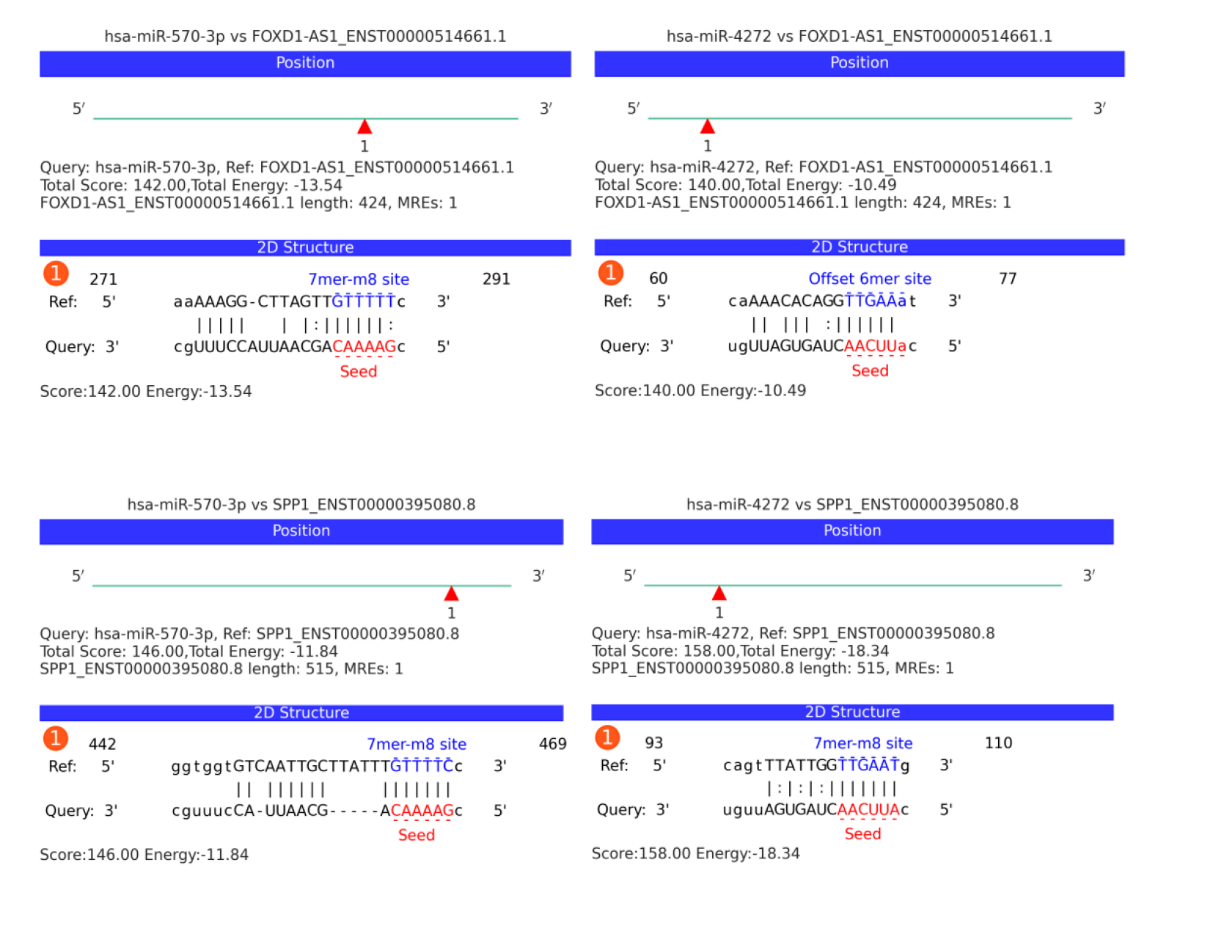
**Figure S2:** According to the Miranda Database, it has been observed that miR-570-3p and miR-4272 possess complementary binding sites inside the lncRNA FOXD1-AS1 and SPP1.

**
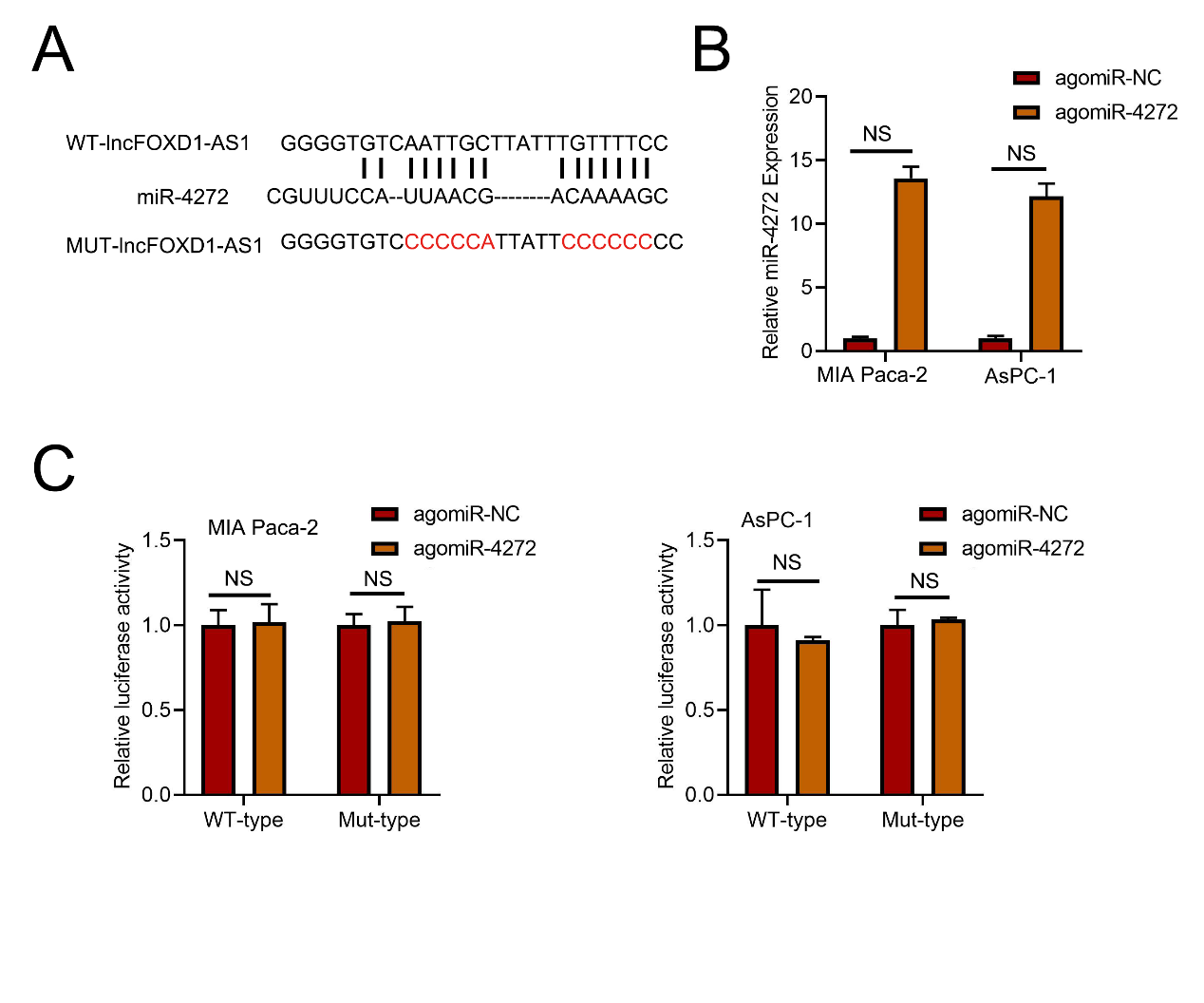
Figure S3** (**A**) The implementation of bioinformatics analysis facilitated the identification of the specific regions where miR-4272 binds to the lncRNA FOXD1-AS1. (**B**) The expression study of miR-4272 was conducted through the RT-qPCR assay. (**C**) On the designated PC cell lines, luciferase reporter assays were performed.

**
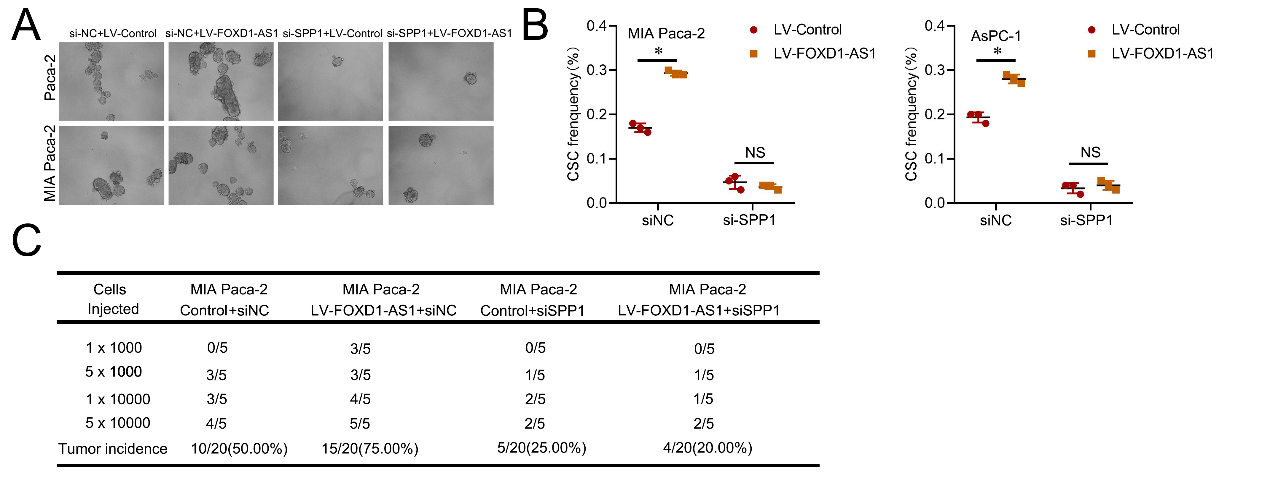
Figure S4 A:** Cells overexpressing lncRNA FOXD1-AS1 and control PC cells were transfected with siRNA targeting SPP1 or NC and subsequently underwent spheroid formation. **B.** After transfecting SPP1 siRNA or negative control, lncRNA FOXD1-AS1 overexpression cells and control PC cells were put through an *in vitro* limiting dilution test. **C.** The in vivo limiting dilution assay was performed on lncRNA FOXD1-AS1 overexpression cells and control PC cells transfected with SPP1 siRNA or NC.

**
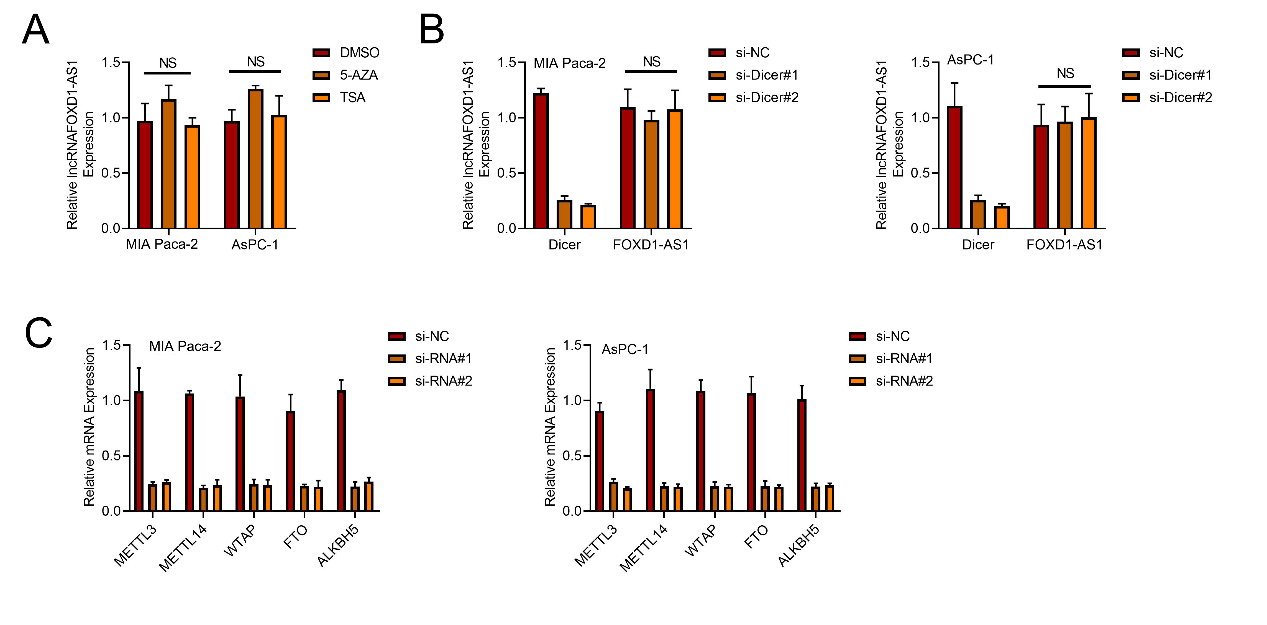
Figure S5: A.** After treating PC cells with DMSO, 5-AZA, or TSA as directed, the lncRNA FOXD1-AS1 level was measured by qPCR. **B.** The level of lncRNA FOXD1-AS1 was analyzed by qPCR in pancreatic cancer cells transfected with si-Control or si-Dicer as specified. **C.** qPCR was performed on pancreatic cancer cells transfected with the designated siRNA (si-Control, si-ALKBH5, si-WTAP, si-METTL14, si-METTL3, or si-FTO).

**
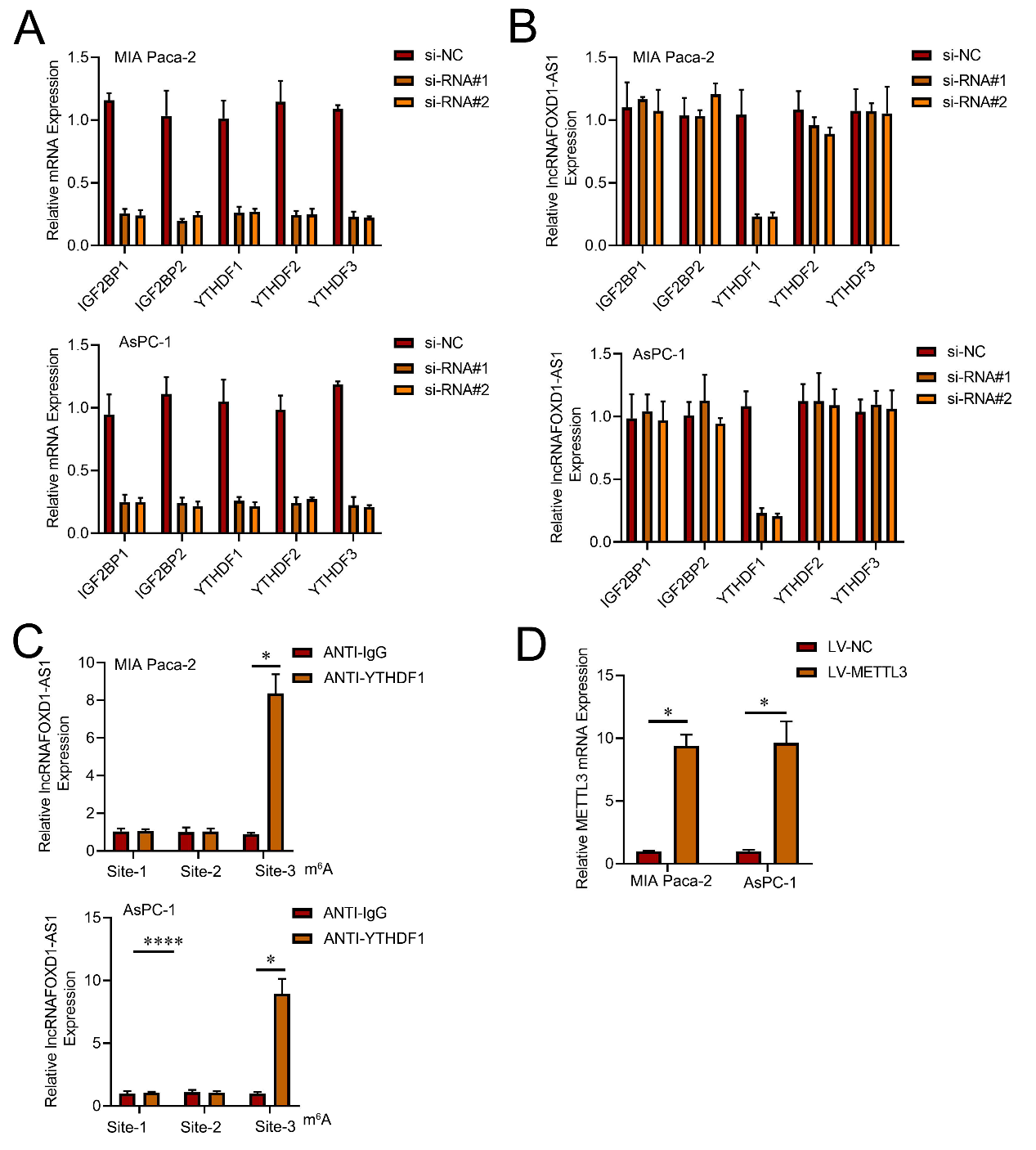
Figure S6: A.** The PC cells were transfected with specific siRNA molecules targeting control siRNA, YTHDF-1/-2/-3, or IGF2BP-1/-2/-3. Subsequently, a qPCR assay was performed on these transfected cells. **B.** The expression level of lncRNA FOXD1-AS1 in PC cells that were transfected with specific siRNAs targeting control siRNA, YTHDF-1/-2/-3, or IGF2BP-1/-2/-3 a was measured using qPCR test. **C.** The m^6^A methylation level in PC cells by m^6^A-qPCR. **D**.qPCR analysis of lncRNA FOXD1-AS1 level in indicated PC cells.

**
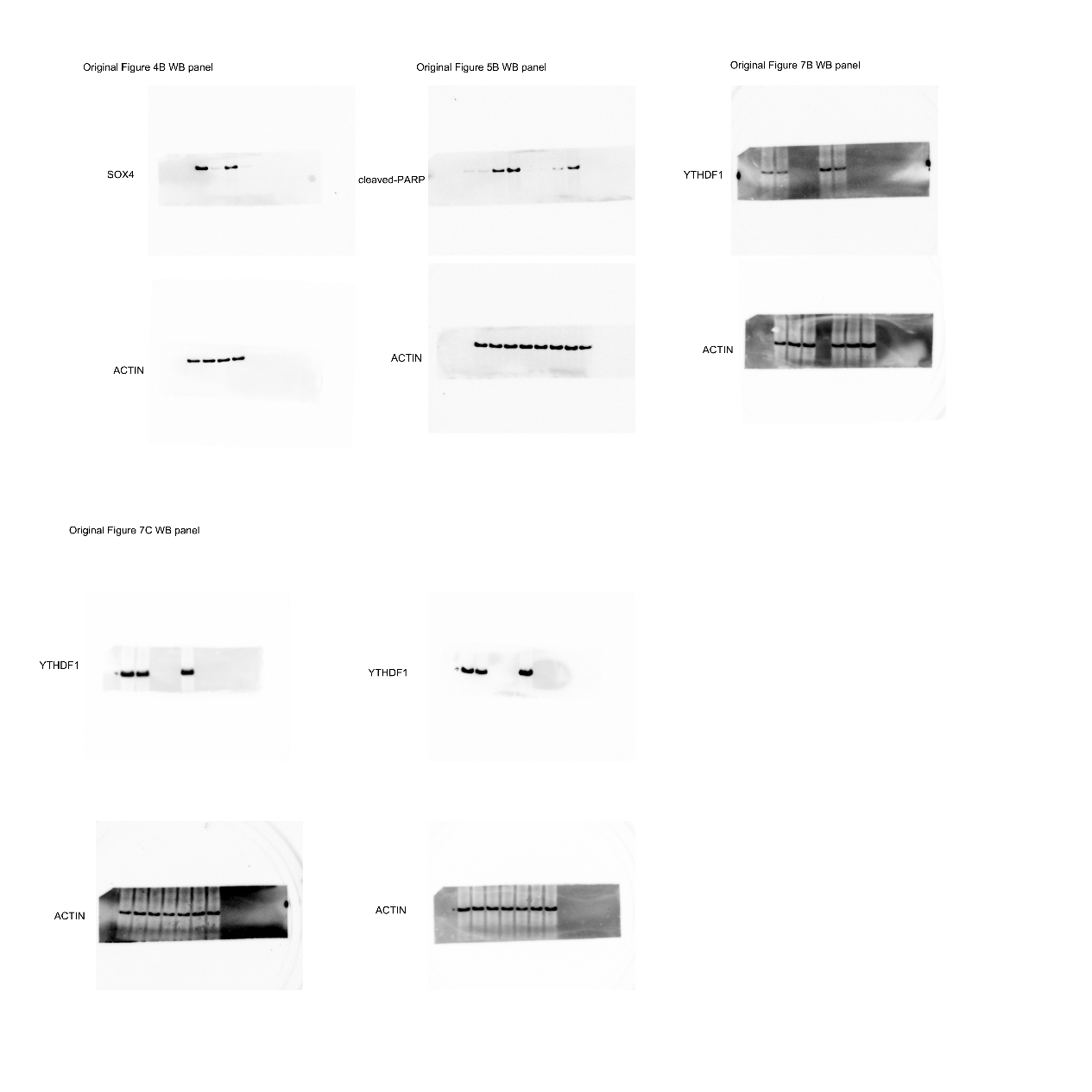
Figure S7 Oringal wb panel**

**Table S1: Clinicopathologic Characteristics of 30 Pancreatic Cancer Patients**

| **Characteristics** | **Number (n=30)** | |
| --- | --- | --- |
| **Age(year)** | ≤50 | 25 |
|  | >50 | 10 |
| **Gender** | Male | 30 |
|  | Female | 5 |
| **Tumor size(cm)** | ≤5 | 9 |
|  | >5 | 26 |
| **Tumor number** | Single | 12 |
|  | Multiple | 23 |
| **Portal vein tumor thrombus** | Yes | 5 |
|  | No | 30 |
| **Encapsulation** | Complete | 12 |
|  | None | 23 |
| **Pathologic satellite** | Yes | 16 |
|  | No | 19 |
| **TNM** | I-II | 12 |
|  | III-IV | 23 |

**Reference**

**1.** Chen S, Zhang Z, Zhang B, Huang Q, Liu Y, Qiu Y, Long X, Wu M, Zhang Z. CircCDK14 Promotes Tumor Progression and Resists Ferroptosis in Glioma by Regulating PDGFRA. Int J Biol Sci. 2022 Jan 1;18(2):841-857.

**2.** Wei WT, Nian XX, Wang SY, Jiao HL, Wang YX, Xiao ZY, Yang RW, Ding YQ, Ye YP, Liao WT. miR-422a inhibits cell proliferation in colorectal cancer by targeting AKT1 and MAPK1. Cancer Cell Int. 2017; 17:91.
